# Dealing With Assumptions and Sampling Bias in the Estimation of Effective Population Size: A Case Study in an Amphibian Population

**DOI:** 10.1111/eva.70015

**Published:** 2024-09-13

**Authors:** Karen Cox, Sabrina Neyrinck, Joachim Mergeay

**Affiliations:** ^1^ Research Institute for Nature and Forest (INBO) Geraardsbergen East Flanders Belgium

**Keywords:** effective population size, linkage disequilibrium, neighbourhood size, *Rana arvalis*, sibship, spatially structured population

## Abstract

Accurately estimating effective population size (*N*_e_) is essential for understanding evolutionary processes and guiding conservation efforts. This study investigates *N*_e_ estimation methods in spatially structured populations using a population of moor frog (*Rana arvalis*) as a case study. We assessed the behaviour of *N*_e_ estimates derived from the linkage disequilibrium (LD) method as we changed the spatial configuration of samples. Moor frog eggs were sampled from 25 breeding patches (i.e., separate vernal ponds, ditches or parts of larger fens) within a single population, revealing an isolation‐by‐distance pattern and a local spatial genetic structure. Varying buffer sizes around each patch were used to examine the impact of sampling window size on the estimation of effective number of breeders (*N*_b_). Our results indicate a downward bias in LD *N*_b_ estimates with increasing buffer size, suggesting an underestimation of *N*_b_. The observed bias is attributed to LD resulting from including genetically divergent individuals (mixture‐LD) confounding LD due to drift. This emphasises the significance of considering even subtle spatial genetic patterns. The implications of these findings are discussed, emphasising the need to account for spatial genetic structure to accurately assess population viability and inform conservation efforts. This study contributes to our understanding of the challenges associated with *N*_e_ estimation in spatially structured populations and underscores the importance of refining methodologies to address population‐specific spatial dynamics for effective conservation planning and management.

## 1 Introduction

The estimation of effective population size (*N*_e_) is of great importance not only for evolutionary biologists but also for conservationists and natural resource managers who aim to monitor and predict population viability (e.g., Allendorf, Luikart, and Aitken 2013; Frankham, Briscoe, and Ballou 2010). With knowledge of *N*_e_ it is possible to assess the rate of random genetic drift, and obtain insight into the three remaining evolutionary forces, namely mutation, gene flow, and selection (Waples 2010). An estimate of *N*_e_ serves as an indicator of genetic stability within a population, with a smaller *N*_e_ leading to an increased rate of genetic erosion (Hoban et al. 2020). This inverse relationship means that as *N*_e_ decreases, the rates of inbreeding and genetic diversity loss rise significantly. However, effective population size is inherently complex in nearly every aspect (Charlesworth 2009). Its benchmark lies in an idealised population at mutation‐drift equilibrium of constant size, closed to immigration, discrete generations and random mating—a scenario rarely met in natural populations (Wright 1931). Methods have been developed to accommodate various violations of assumptions (e.g., spatial structure, gene flow, non‐equilibrium, overlapping generations), but this introduces a suite of different types of *N*_e_ that only make sense in a spatially and temporally explicit context, and which can differ strongly from each other (Tenesa et al. 2007; Ryman, Laikre, and Hössjer 2019; Nadachowska‐Brzyska, Konczal, and Babik 2022; Waples 2022). Nonetheless, even these require simplified, unrealistic assumptions.

Directly calculating *N*_e_ from demographic data is challenging, which is why genetic methods are typically used. Particularly popular are the ‘Single‐sample’ *N*_e_ estimators that offer a pragmatic approach by relaxing temporal sampling requirements (Waples and Do 2010). Among the most widely used single‐sample methods is a bias‐adjusted version of the linkage disequilibrium (LD)‐based method of Hill (1981) (Waples 2006, 2024; Waples and Do 2008). Despite its utility, this estimator, and any other available at this moment, operates under simplifying assumptions, with its sensitivities extensively assessed in earlier studies (e.g., Neel et al. 2013; Waples, Antao, and Luikart 2014; Nunney 2016). In natural populations, gene flow and spatial structure pose challenges for the estimation of effective population size. Gene flow and sampling beyond any panmictic entity creates a sample that violates the assumptions of the method; this gives rise to LD as a result of incorporating genetically divergent individuals (mixture‐LD), whereas the model assumes all LD is the result of genetic drift. The result is an underestimation of *N*_e_. On the other hand, when a sample is drawn from a broader parent pool compared to a sample derived only from local breeders, this could reduce the apparent drift and inflate the *N*_e_ estimate (Waples and England 2011). Whether LD is positively or negatively affected depends on the magnitude of both confounding factors. The challenge becomes particularly important in populations occurring over spatial extents that greatly exceed the individual dispersal distance and which experience internally isolation by distance. No matter how you sample such a population, you always violate either the assumption of random mating or the assumption that the sample represents the population. These are typically called ‘continuous populations’ with examples among many taxa, such as the mountain goat (*Oreamnos americanus*) (Shirk and Cushman 2014), the Florida scrub‐jay (*Aphelocoma coerulescens*) (Aguillon et al. 2017) or even yellow fever mosquitoes (*Aedes aegypti*) (Jasper et al. 2019).

In relation to such continuous populations, Wright (1946) introduced the concept of the neighbourhood size (*N*_n_), which represents the local effective size within which mating can be considered random. Assuming Gaussian dispersal, this is approximated by

$$N_{n}=\pi {\left(2\sigma \right)}^{2}D$$

with *D* the density of effective individuals and *σ* the mean dispersal distance between parent and offspring. The breeding window is then the area within which mating can still be considered largely random, and is the area of a circle with radius 2*σ*.

Neel et al. (2013) confirmed through simulations the downward bias of mixture‐LD on effective number of parents (*N*_b_), where estimates of *N*_b_ were assessed with differing breeding window and sampling window sizes in a continuously distributed population. As expected from theory (Wright 1946), *N*_b_ was nearly equal to the genetic neighbourhood size when samples were taken within the breeding window. The larger the sampling window relative to the breeding window, the slower the increase in *N*_b_ until an asymptote was reached corresponding to a substantial lower *N*_b_ estimate than the true total *N*_b_. Besides continuous populations with mating being restricted by distance (i.e., isolation‐by‐distance), there are other forms of natural populations that suffer from this same principle. Many pond‐breeding amphibians form spatially structured populations, which encompass but are not restricted to classical metapopulations (Thomas and Kunin 1999; Smith and Green 2005; Revilla and Wiegand 2008). They consist of a set of local populations occupying distinct habitat patches that are connected through dispersal. Although most dispersal can still be local, it also can be dependent on distance, on the presence and density of conspecifics, the number and size of habitat patches or subpopulations and their specific location (for a review, see Cayuela et al. 2020).

In this study, we evaluated empirically the behaviour of estimates of *N*_e_ using the LD‐method when incrementally more breeding patches within a spatially structured but largely continuous population of moor frog (*Rana arvalis* Nilsson 1842) were included in a single sample. This species uses its aquatic habitat for breeding during a short period in early spring. The majority of the moor frog's time is spent on land, mostly within 400 m of the breeding pond (Kovar et al. 2009). Nonetheless, the species can migrate and disperse for longer distances, 1 to several kilometres (Vos et al. 2001; Kovar et al. 2009), which exceed the dimensions of our study site. Here, we maximised sampling of a single cohort, which would allow us to obtain an estimate of the census and effective number of parents. As an iteroparous species with overlapping generations, the LD estimate of *N*_e_ corresponds more with the effective number of parents or breeders *N*_b_ rather than the effective size (Waples 2005; Waples, Antao, and Luikart 2014). We compared *N*_b_ estimated for different sampling windows with the number of potential breeding adults, and assessed if similar patterns emerged as in the simulation study of Neel et al. (2013) and how the results connect with the spatial structure of the population.

## 2 Materials and Methods

### 2.1 Study Area and Sampling

The study site of c. 200 ha is part of the nature reserve and military domain ‘Klein Schietveld’ in Kapellen near Antwerp, Belgium (51.358 N, 4.495 E; Figure S1). This 19th‐century heathland consists of wet heathland and fens, dry heathland, inland dunes and sparse grasslands. In March 2017, heathland pools, fens and temporary ponds were screened for the presence of egg clutches possibly belonging to moor frogs. In total, eggs were sampled in 26 locations where clusters of clutches were found (Figure S1). These locations consisted of separate vernal ponds, ditches or parts of larger fens. We call these locations ‘breeding patches’ from now on. In each breeding patch, up to 50 intact and distinguishable clutches were sampled and three eggs per clutch were taken. The total number of clutches were counted at the time of sampling and a week before by two persons. In total, 493 clutches were sampled (Table 1). In two breeding patches, eggs were already hatched; therefore, 30 larvae were sampled instead. In one other breeding patch, HK1, only one clutch was sampled while an adult female moor frog was depositing her eggs. These samples served as a reference of moor frogspawn in the species identification (see the following section). The number of clutches was not counted at that patch. The eggs and tadpoles were stored in pond water in a refrigerator until DNA extraction (maximally a few days after sampling).

**TABLE 1:** Number of egg clutches counted and sampled in Klein Schietveld with the number of clutches assigned to *Rana arvalis* as determined using RFLP analysis developed by Palo and Merilä (2003). Estimates of effective number of breeders using two methods are shown for each patch.

Patch ID	*N* counted	*N* sampled (*N* larvae)	N *R. arvalis*	% *R. arvalis* clutches	LD ${\hat{N}}_{b}$ (95% CI)	Sibship ${\hat{N}}_{b}$ (95% CI)
HK1	NA	1	1	NA		
01	13	13	13	100	16.1 (10.3–28.0)	26 (14–52)
02	70	31	3	10	6.4 (2.4–32.7)	7 (2–41)
1	85	9 (30)	3	3	2.9 (2.1–6.9)	10 (4‐∞)
10	37	17	17	100	31.8 (21.7–51.5)	29 (17–54)
11	16	12	12	100	24.6 (16.8–40.2)	15 (7–34)
13	47	18	16	89	29.8 (20.9–46.1)	31 (18–55)
14	35	14	14	100	22.3 (14.7–37.7)	24 (13–47)
16	60	37	10	27	21 (12.9–41.6)	18 (10–39)
17	35	21	7	33	8.2 (5.2–13.0)	17 (9–39)
18	51	51	50	98	54.0 (40.7–74.1)	95 (69–129)
19	25	21	21	100	48.7 (31.5–88.6)	33 (20–56)
20	4	4	4	100	2.8 (2.0–7.0)	9 (4–28)
21	25	18	8	41	15 (9.5–26.5)	17 (8–41)
22a	100	13	12	92	55.7 (31.1–172.2)	19 (10–39)
22b	70	17	16	94	31.0 (22.0–47.1)	29 (17–51)
22c	38	15	15	100	18.5 (13.2–27.2)	36 (20–71)
24	500	0 (30)	0	0		
25	50	25	25	100	41.6 (32.3–55.8)	45 (29–69)
3	14	14	14	100	19.6 (12.7–33.6)	28 (14–60)
30	42	36	0	0		
31	50	19	18	95	34.8 (23.3–58.1)	38 (24–64)
32	3	3	3	3	16.9 (3.8‐∞)	5 (2–32)
4	40	21	21	100	40.3 (24.5–80.5)	41 (26–68)
5	9	9	9	100	38.3 (18.3–278.8)	12 (6–28)
6	85	41	41	100	75.4 (50.2–128.2)	64 (46–95)
7	17	13	13	100	21.2 (15.6–30.3)	22 (12–43)
Sum	1521	493 (60)	366		676.9	670
KS					381.3 (290.1–519.1)	615 (541–691)

*Note:* For each sampling location (Patch ID), the table shows the number of counted/estimated number of egg clutches (*N* counted), the number of sampled clutches (*N* sampled) and the number of sampled larvae between parentheses (*N* larvae) when applicable, the number of clutches of those sampled identified as from *R. arvalis* (N *R. arvalis*), the percentage *R. arvalis* clutches (% *R. arvalis* clutches) among the sampled clutches, the estimates of effective number of breeders using the linkage disequilibrium method (LD ${\hat{N}}_{b}$), the sibship method (Sibship ${\hat{N}}_{b}$), and their corresponding 95% confidence intervals (95% CI). Values of ${\hat{N}}_{b}$ are also given when all samples of the population of Klein Schietveld (KS) are included in the calculation.

### 2.2 DNA Extraction and Species Identification

DNA extraction was performed on two eggs per clutch. The jelly coats were first removed using a scalpel. DNA was extracted from the embryo's using DNeasy Blood & Tissue Kit (Qiagen) with a lysis step of 1 hour and eluted in 70 μL AE buffer (elution performed twice). The same kit was used for the larvae and DNA was eluted in 140 μL AE buffer. The integrity of DNA of 10% of the samples was assessed on 1% agarose gels, while the DNA concentration of all tissue samples was measured with Quant‐iT Picogreen dsDNA Assay Kit (Invitrogen, Thermo Fisher Scientific) using a Synergy HT plate reader (BioTek).

In order to make the distinction between samples from two different species, *Rana arvalis* and *R. temporaria*, DNA from one egg per clutch and from all larvae was analysed with the RFLP method of Palo and Merilä (2003). Both species live in sympatry and cannot easily be identified based on egg and larval morphology. A 605‐bp fragment of cyt‐b was amplified with primers L14850 (5'‐TCTCATCCTGATGAAACTTTGGCTC; Tanaka, Matsui, and Takenaka 1994) and H15410 (5'‐GTCTTTGTAGGAGAAGTATGG; Tanaka, Matsui, and Takenaka 1996), and digested with restriction enzyme *Hae*III. PCR amplification was performed using 2 μL undiluted DNA in a total volume of 20 μL with 1x PCR buffer, 250 μM of each dNTP, 0.1 μM of each primer, 1 U Taq polymerase and 1.5 mM MgCl_2_. PCR products were checked on 2% agarose gels. The PCR conditions described by Palo and Merilä (2003) were slightly adjusted: initial step of 3 min at 94°C, 35 cycles of 94, 49 and 72°C 30 s each, and a final elongation step of 5 min at 72°C. The restriction digests were performed in a total volume of 20 μL with 10 μL PCR product, 2 μL 10x Buffer R (Thermo Scientific), 1 μL *Hae*III (10 U/μL) and 7 μL MQ water, with an incubation of 2 h at 37°C. Fragments (10 μL) were loaded on 2% agarose gels containing 2 μL DNA Gel Loading Dye (6×; Thermo Scientific) and a Invitrogen 100 bp DNA size standard ladder. The eggs taken from a clutch at HK1 were used as a positive reference for moor frog (see previous section). On the basis of species identification results, the percentage of moor frog clutches present in each breeding patch was calculated.

### 2.3 Genotyping

Only samples identified as *Rana arvalis* were genotyped using 19 microsatellite markers in two simplex and two multiplex reactions (Brys, Cox, and Neyrinck 2020; Mergeay et al. 2020). The PCR amplifications were performed in a solution of 5 μL Multiplex PCR Master Mix (Qiagen), 0.05, 0.20, 0.40 or 0.60 μL of each primer set (10 μM; see Brys, Cox, and Neyrinck 2020), 1 μL DNA with a concentration of c. 5 ng/μl, and was adjusted to a final volume of 10 μL by adding diethylpyrocarbonate (DEPC) water (Sigma‐Aldrich, Overijse, Belgium). The cycling profile for the first multiplex consisted of 15 min at 95°C followed by 35 cycles with 30 s at 95°C, 45 s at 50°C and 45 s at 72°C, and with an elongation step of 10 min at 72°C. We used the same PCR profile for multiplex 2, but with an annealing temperature of 55°C. A touchdown procedure was used for marker RA13 with 15 min at 94°C, 22 cycles of 30 s at 94°C, 30 s starting at 60°C and reducing 0.5°C each cycle, 30 s at 72°C followed by 15 cycles of 30 s at 94°C, 30 s at 50°C and 30 s at 72°C, ending with 10 min at 72°C. For marker RtU4, the same conditions were used as for RA13, except for the touchdown cycles; here, 35 cycles were performed at 94, 46 and 72°C 30 s each. Each reaction ended with 15 min at 4°C after which the PCR product was kept at 15°C. PCR products from multiplex reactions were diluted 1:10 before running them on a ABI 3500 Genetic Analyzer (Applied Biosystems). PCR products from simplex reactions were analysed in poolplex using a dilution of 1:50. Genotypes were analysed with the GeneMapper v4.1 software package with fragment sizes based on GeneScan 600 LIZ Size Standard (Applied Biosystems). Negative controls were included in each 96‐well PCR to allow for detection of reagent contamination. Reproducibility was evaluated using 3% blindly replicated samples, two to five times within and across well plates. One reference sample was further added to each well plate. Samples with genotypes for less than 50% of the loci were reanalysed or replaced with genotypes of eggs of the same clutch where possible.

We tested for the presence of null alleles, for possible deviations from Hardy–Weinberg equilibrium (HWE), and for systematic LD between pairs of loci. All tests were performed with genepop v4.6 R package (Rousset 2008). We implemented the Bonferroni correction for multiple testing with a nominal level of 5%. Besides using the maximum likelihood method following the expectation maximisation algorithm of Dempster, Laird, and Rubin (1977), implemented in genepop, to assess the presence of null alleles, we also used ML‐Null Freq (Kalinowski and Taper 2006). To assure no Wahlund effect could influence the test results for HWE, we performed tests at the patch level. Eggs from the same clutch always constitute half or full siblings, since clutches can be fertilised by multiple male *R. arvalis* (Knopp and Merilä 2009). Sampling more related individuals than by chance can bias structure and genetic diversity results (Goldberg and Waits 2010; Sánchez‐Montes et al. 2017; O'Connell et al. 2019). As we used at most two genotypes per clutch in our analyses, no large imbalance in family sizes among samples existed. However, significant deviation from HWE can be caused by sampling families or multiple cohorts (Hansen, Nielsen, and Mensberg 1997; Jankovic, vonHoldt, and Rosenberg 2010). We, therefore, repeated the tests after excluding one full sibling per pair. Full‐sib families were identified using COLONY v2.0.6.5 following a maximum likelihood approach (Wang 2004; Jones and Wang 2010). The method also takes error rate and allelic drop out in consideration. The latter was estimated based on replicated genotypes with the software Pedant v1.0 (Johnson and Haydon 2007). We used 0.0001 as the error rate for each locus since the rate based on replicates was mostly 0 and on average 0.01 per locus. The following settings in COLONY were used: full likelihood method with high precision, random mating, polygamous mating system and three runs of medium length. Information that eggs from the same clutch share the same mother was included as a prior. Only pairs with a probability higher than 0.90 were considered to be full siblings. Structure and differentiation analyses were done without full siblings unless mentioned otherwise.

### 2.4 Spatial Structure

In order to detect a potential spatial genetic structure, we performed a spatial principal component analysis (sPCA), which is independent from Hardy–Weinberg assumptions or LD (Jombart et al. 2008), using the package adegenet v2.1.10 (Jombart 2008) in R v4.3.0 (R Core Team 2023). The sPCA approach utilises allele frequencies and accounts for their genetic variability and spatial autocorrelation, calculated with Moran's *I* (Moran 1948, 1950) on the basis of a connection graph. We used the neighbourhood‐by‐distance graph with a minimum distance of 0 and a maximum distance of 1 km, which corresponds to the maximum distance observed for *R. arvalis* during spring migration (Kovar et al. 2009). The presence of a global and local spatial structure was tested with 999 Monte Carlo permutations as described by Montano and Jombart (2017). Global and local structures differ in their patterns of spatial autocorrelation. Global structures show positive spatial autocorrelation, meaning genetic similarities increase with geographic proximity, forming clear spatial groups or gradients (e.g., isolation‐by‐distance). In contrast, local structures exhibit negative spatial autocorrelation, indicating stronger genetic differences among nearby individuals than among those chosen randomly. Essentially, global patterns highlight broad genetic distinctions across regions, while local patterns emphasise more pronounced genetic differences at a finer spatial scale.

We further calculated Moran's*I* among pairs of individuals and performed a spatial autocorrelation analysis with SPAGeDi v1.5 (Hardy and Vekemans 2002). The results were used to find the distance over which individuals are not genetically independent, which could help to identify the size of the breeding window. We chose 10 distance intervals to obtain sufficiently small distance classes with an approximately constant number of pairwise comparisons in each interval. Significant deviation of spatial autocorrelation from a random distribution of genotypes was tested for each distance class with 10,000 random permutations of individual locations.

Global and pairwise *F*_ST_ of Weir and Cockerham (1984) were calculated among breeding patches with at least five samples, with corrected 95% confidence intervals using 1000 bootstrap iterations with the R package diveRsity v1.9.90 (Keenan et al. 2013).

### 2.5 Sampling Windows

To evaluate the effect of enlarging the sampling window on the estimate of *N*_b_ (${\hat{N}}_{\text{b}}$), we created buffers around each sampled breeding patch with different radii (Figure S2). The radius changed from 100 m to 1 km with steps of 100 m. Certain buffers contained the same patches. Such duplicates were removed. When duplicates were found among different buffer sizes, the smallest was retained. A final sampling window included all breeding patches of the study area and corresponded with a buffer radius of 1.5 km for most of the patches. Out of 126 unique combinations of breeding patches within the different buffers, one combination included only the HK1 patch, resulting in just one clutch. This combination was excluded from further data analysis. Calculations were done with R packages tidyverse v2.0.0 (Wickham et al. 2019), sp. 1.6‐0 (Pebesma and Bivand 2005; Bivand, Pebesma, and Gomez‐Rubio 2013) and rgeos v0.6‐2 (Bivand and Rundel 2023).

### 2.6 Effective Number of Breeders

We estimated the number of parents (*N*_P_) of the eggs included in the genetic analysis and for each sampling window (i.e., buffer size) using the sibship method of COLONY, as described earlier. Since female moor frogs lay one clutch per year (Räsänen et al. 2008; Richter‐Boix et al. 2011; Schmeller and Merilä 2007), the number of clutches was applied as a proxy for the number of mothers of the sampled eggs. The average ratio of the number of mothers to *N*_P_ estimated with COLONY was used to estimate the sex ratio of the population. To calculate the number of potential breeding adults (*N*_A_) within each sampling window, we applied this sex ratio to the number of counted moor frog clutches. This number equals all the counted clutches corrected by the percentage of analysed clutches identified as moor frog frogspawn (Table 1). Since we counted most of the frogspawn on site, the number could be considered as an approximate census of breeding females of this breeding season, though still with a downward bias.

The effective number of breeders was estimated using the sibship method of COLONY and the LD method LDNe (Waples and Do 2008) implemented in NeEstimator v2.1 (Do et al. 2014). The jackknife method was used to estimate 95% confidence intervals across loci; a threshold for minimum allele frequency was set at the 0.02 and 0.05 thresholds (Waples and Do 2010). Because results for both thresholds were very similar (results not shown), we only used 0.02 as a minimum allele frequency threshold for further analysis.

The sibship *N*_e_ is estimated based on frequencies of full‐ and half‐sib dyads within a sample (eq. 10 in Wang (2009)):

$$\frac{1}{N_{e}}=\frac{1+3\alpha }{4}\left(Q_{\mathrm{HS}}+2Q_{\mathrm{FS}}\right)-\frac{\alpha }{2}\left(\frac{1}{N_{m}}+\frac{1}{N_{f}}\right)$$

where *α* quantifies the deviation from HWE or the correlation of genes within individuals relative to the genes taken at random from the population (Wright 1969), where *Q*_HS_ and *Q*_FS_ present the frequencies of half‐ and full‐sib dyads, respectively, and with the number of breeding males, *N*_m_, and females, *N*_f_. Since we collected two eggs per clutch, frequencies of half and full sibs did not differ much among sites. Considering *α* is close to zero under random mating, the resulting sibship ${\hat{N}}_{b}$ will largely correspond with the number of parents of the sampled eggs.

We further calculated the inbreeding coefficient *F*_IS_ (Nei 1987) using the R package hierfstat v0.5‐11 (Goudet and Jombart 2022). We compared with *F*_IS_ calculated after keeping one individual per full‐sib dyad.

Calculations were done for each breeding patch (with at least six individuals) and for each buffer radius. We compared the different ${\hat{N}}_{b}$ with sample and buffer size. Estimates of *N*_b_ without finite confidence limits were not used in further analyses. The ratio of ${\hat{N}}_{b}$ to *N*_A_ was examined using the different buffer radii. We further investigated the behaviour of *F*_IS_ to an increasing buffer radius.

To get a sense of the metapopulation *N*_b_ (meta‐*N*_b_), different approaches were used and compared. Assuming that genetic structure is the only deviation from a Wright–Fisher population, meta‐*N*_e_ = *N*/(1−*F*_ST_) Wright (1931, 1943), with *N* the total population size. Spieth (1974) showed that *N* = ∑*N*_n_ in an ideal population, so we can rewrite meta‐*N*_e_ = ∑*N*_n_/(1−*F*_ST_). This model assumes non‐random mating given a spatial structure of semi‐isolated island subpopulations with random mating within. The island model was used with local ${\hat{N}}_{b}$ estimated at the breeding patch‐level on the one hand, and at the level of groups of breeding patches defined by the sPCA results on the other hand. Global *F*_ST_, included in the model, was calculated for both structure levels. We compared both estimates with total sums of local ${\hat{N}}_{b}$.

### 2.7 Random Sample

On the basis of genetic structure results, we estimated the radius of the breeding window. To evaluate whether we could approximate the neighbourhood ${\hat{N}}_{b}$ with minimal sampling effort, we placed buffers with this radius randomly within the study area. We considered the local structure as unknown as this would entail a widespread sampling scheme. We selected 10 buffers that included at least 50 samples and more than one breeding patch. Within each buffer, 50 samples were selected randomly; this was repeated 10 times in each buffer. For each buffer and set of samples LD ${\hat{N}}_{b}$ was calculated. We then estimated meta‐${\hat{N}}_{b}$ by multiplying this value with the expected number of neighbourhoods.

### 2.8 Ethics Statement

The sampling and handling of the frog eggs and larvae were in line with a derogation on the Decision of the Flemish Government on species protection and species management of 15 May 2009 and later amendments to that legislation (permit numbers ANB/BL/FF‐V15‐00232, ANB/BL/FF‐V15‐00233, ANB/BL/FF‐V15‐00234, ANB/BL/FF‐V15‐00235, ANB/BL‐FF/17‐000035; issued by the Agency for Nature and Forests (ANB)). All applicable institutional and national guidelines for the care and use of animals were followed.

## 3 Results

### 3.1 Species Identification and Genotyping

Egg clutches in 11 of the 26 sampled breeding patches were identified as frogspawn from *R. temporaria* (Table 1). Also, sampled larvae were all of this species. We retained samples of *R. arvalis* for further analysis.

Three markers, Rtempμ4, Rtempμ5 and Rt2Ca2‐22, showed no polymorphism and alleles of locus Rtempμ9 could not be identified unambiguously. Locus RECALQ showed deviations from HWE in 5 breeding patches and proportions of null alleles higher than 0.20 in at least 10 breeding patches when comparing results among methods and with full siblings except one excluded. Also, RlatCa41 deviated from HWE in seven breeding patches and null alleles in at least six patches. Both markers were excluded from further analysis. The remaining 13 microsatellites showed significant departures from HWE in at most two breeding patches and higher proportions of null alleles in maximally one patch. No systematic LD between pairs of loci was detected.

### 3.2 Spatial Structure

**FIGURE 1:**
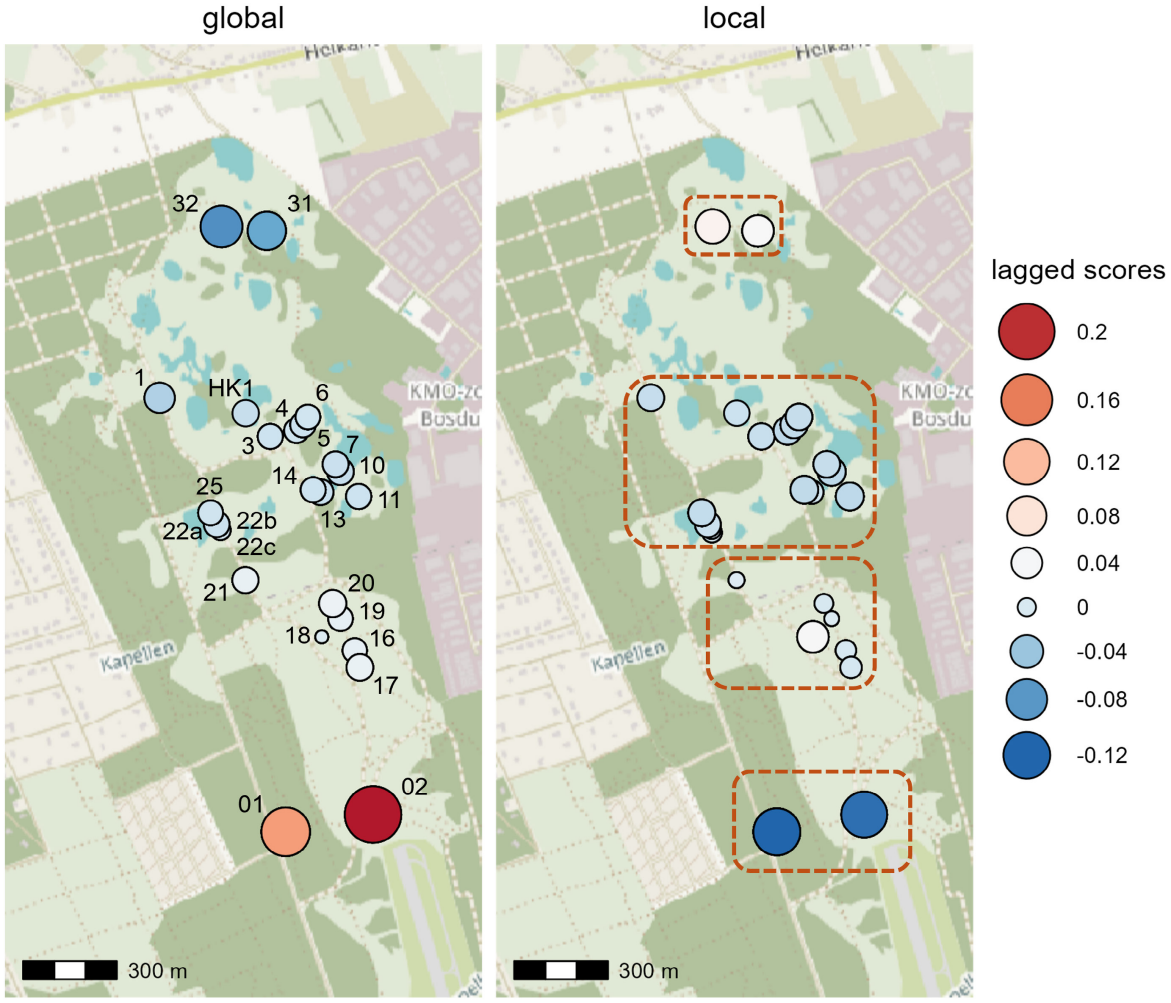
Spatial genetic structure detected with the spatial principal component analysis (sPCA). The lagged scores of the eigenvalues representing the global (left) and local structure (right) were plotted as circles with size and colour scaled relative to their values. Breeding patch IDs are given on the left map; the four groups delineated on the basis of the local structure are in indicated on the right map (dashed outline), corresponding to four local neighbourhoods or breeding windows. Base map: © OpenStreetMap contributors.

**FIGURE 2:**
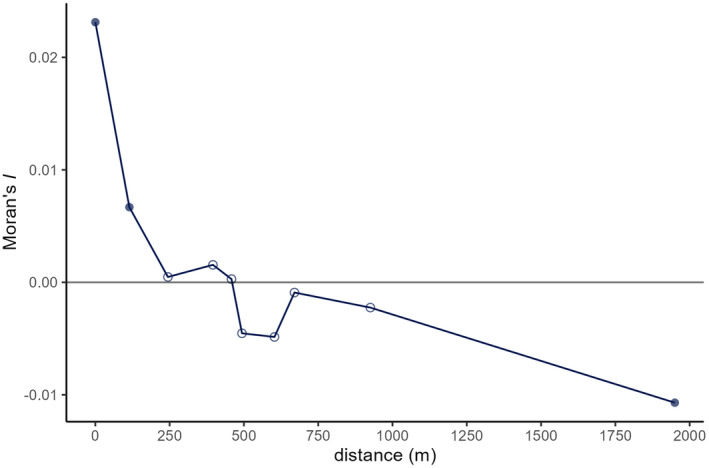
Spatial autocorrelation for 10 distance intervals between samples of moor frog. Each point represents the mean pairwise Moran's *I* of each distance class. Significance of spatial autocorrelation is indicated with full circles (*p* ≤ 0.002) and non‐significance with empty circles.

Results of sPCA included a significant global and local structure (*p* = 0.001). The global pattern, mostly explained by the first positive component, showed a north–south clinal pattern indicative for isolation‐by‐distance (Figure 1). A more subtle though significant local structure (*p* = 0.001), using the first negative component, was found. There were four clusters, each consisting of neighbouring ponds, with a few slightly more distinct ponds (Figure 1). Positive spatial autocorrelation was present up to a maximum distance of 458 m (mean distance of 433 m) (Figure 2). However, it was significant (*p* = 0.002) until a maximum of 114 m (mean of 70 m) was reached.

**FIGURE 3:**
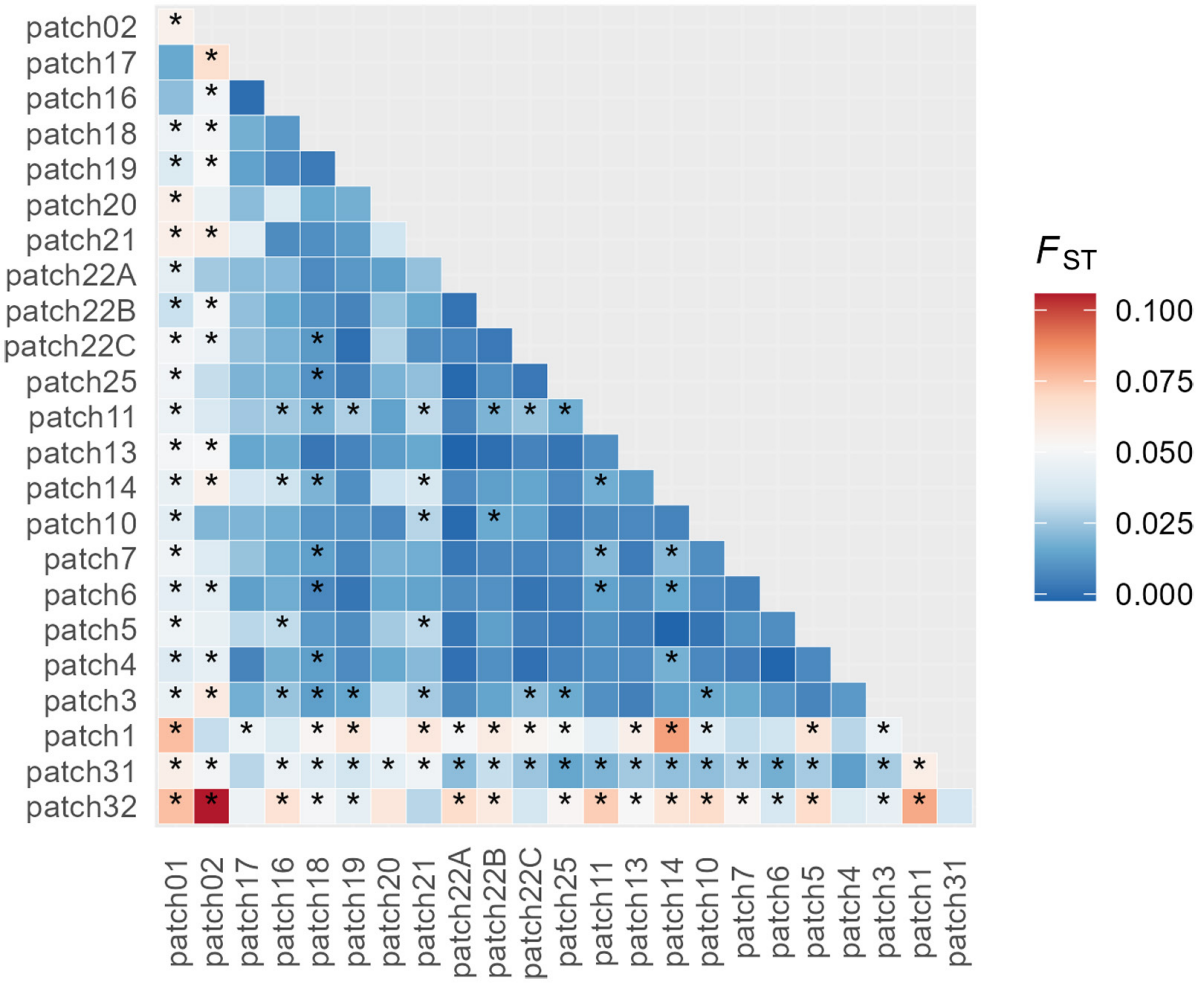
Heatmap of pairwise *F*_ST_ among breeding patches of moor frog. Values significant at the 95% level are indicated with *.

Global *F*_ST_ was 0.0162 (95% CI [0.0110, 0.0218]) and pairwise *F*_ST_ ranged from 0.00 to 0.11 (Figure 3). Though the elevated and significant *F*_ST_ in comparisons involving breeding patches 02, 1 and 32 might be due to low sample sizes (5–6 samples). Geographically more isolated breeding patches (1, 01 and 02, 31 and 32) showed higher differentiation, while there were breeding patches that showed some higher and/or significant *F*_ST_ values even when compared to nearby patches (e.g., breeding patch 11, 21 and 18).

#### 3.2.1 Sampling Window Versus ${\hat{N}}_{b}$

The ratio of the number of mothers to the number of parents *N*_P_ identified with COLONY ranged from 0.34 to 0.62 (mean = 0.48, SD = 0.07) depending on the breeding patches included in the calculations. The mean females:males ratio was thus 48:52. The number of potential breeding adults (*N*_A_) for each of the buffers was obtained on the basis of this mean ratio and on the number of counted clusters corrected by the percentage of moor frog egg clutches. The values of *N*_A_ ranged from 6 to 1510 across all breeding patches and sampling windows.

**FIGURE 4:**
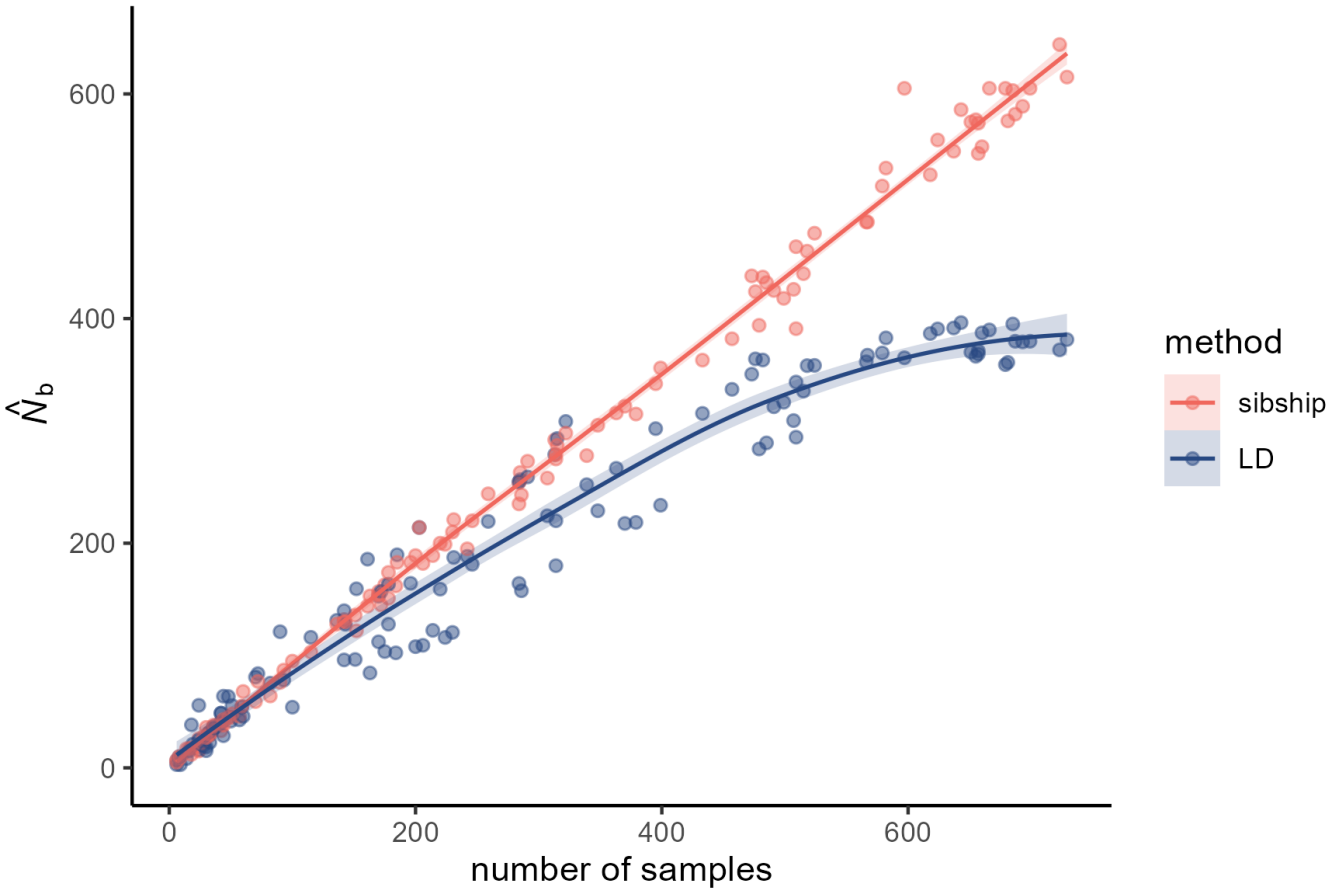
${\hat{N}}_{b}$ obtained using the sibship method (red) and LD method (blue) in relation to sample size depicted using a loess curve with 95% confidence intervals (shaded area).

**FIGURE 5:**
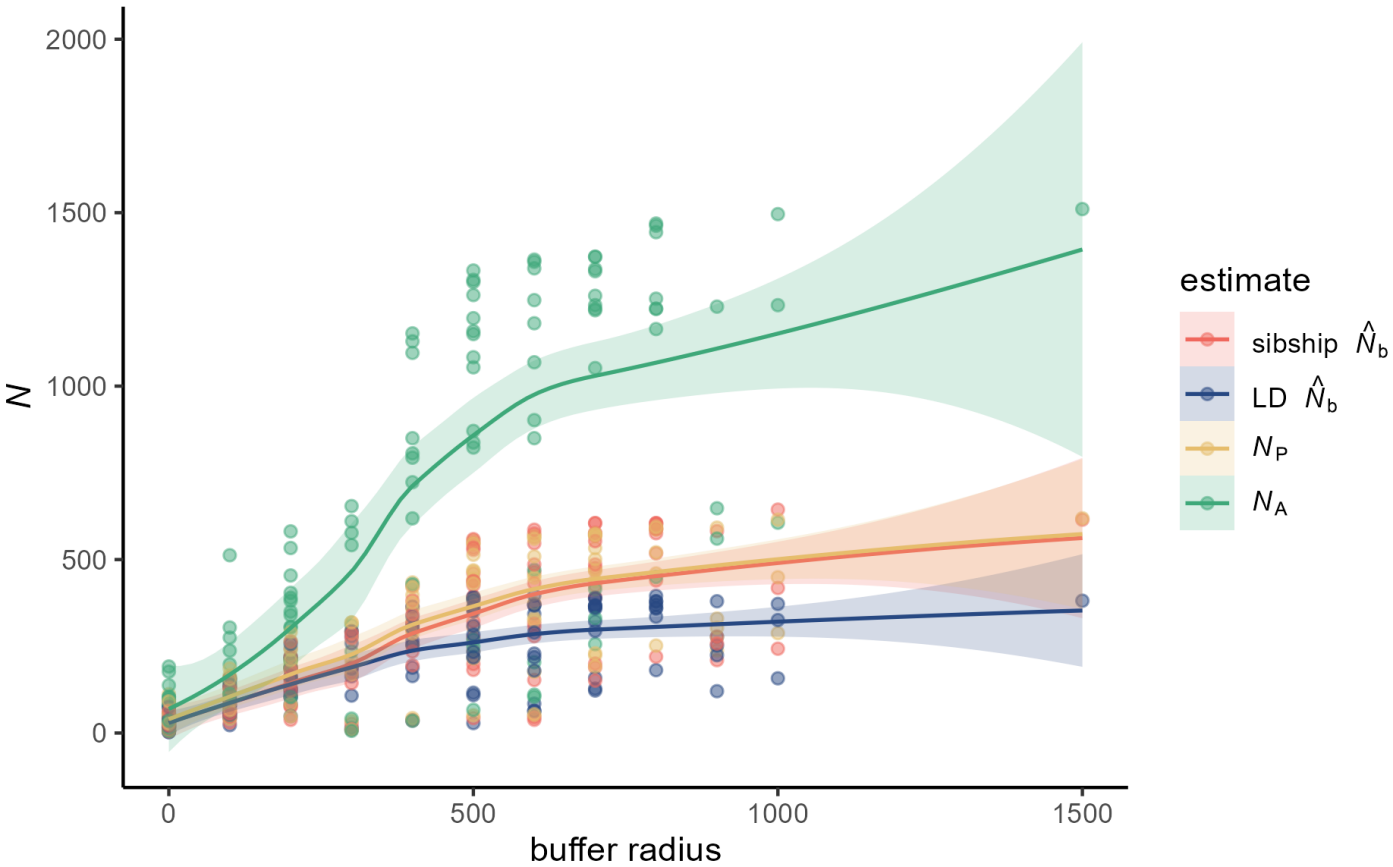
Plot of ${\hat{N}}_{b}$ obtained using the sibship method (red) and LD method (blue), the number of parents (*N*_P_) estimated with the sibship method (yellow) and the number of potential breeding adults (*N*_A_) estimated based on the number of counted clutches and average sex ratio, all in relation to buffer radius, depicted using loess curves with 95% confidence intervals (shaded area).

There were one and two ${\hat{N}}_{b}$ calculated with the sibship and LD method, respectively, without finite upper confidence limits. The ${\hat{N}}_{b}$ per breeding patch and for the entire population when considered as panmictic are provided in Table 1. The ${\hat{N}}_{b}$ of both methods increased with an increasing sample size (Figure 4). This was as expected a near perfect linear relationship for sibship ${\hat{N}}_{b}$, while LD ${\hat{N}}_{b}$ showed a lower increase and a semi‐logarithmic curve; the horizontal asymptote was not yet reached. This was also the case in relation to buffer size, and for both estimates (Figure 5). The LD ${\hat{N}}_{b}$ started off only slightly lower than sibship ${\hat{N}}_{b}$ at the breeding patch level; the difference between both estimates gradually increased with increasing buffer radius, with a clear difference from a buffer radius of 300 m and onward. The increase of LD ${\hat{N}}_{b}$ slowed down at the 400 m radius. The maximum ${\hat{N}}_{b}$ was reached when a considerable number of sampled breeding patches were included, and was 644 (with all breeding patches except patch 02) and 396 (with all patches except 01, 02, 17, 31 and 32) calculated with the sibship and LD method, respectively. Estimates using all available samples were very close to those maximum values (Table 1). Due to our sampling method focusing on an even representation per clutch we actually have no estimate of the variance in reproductive success among parents; the curve of the sibship estimate of ${\hat{N}}_{b}$ versus buffer radius is, therefore, nearly identical to the curve of *N*_P_ estimated with the same method (see material and methods). The number of potential breeding adults *N*_A_ is evidently related to the latter, though it is much higher than both ${\hat{N}}_{b}$ estimates.

**FIGURE 6:**
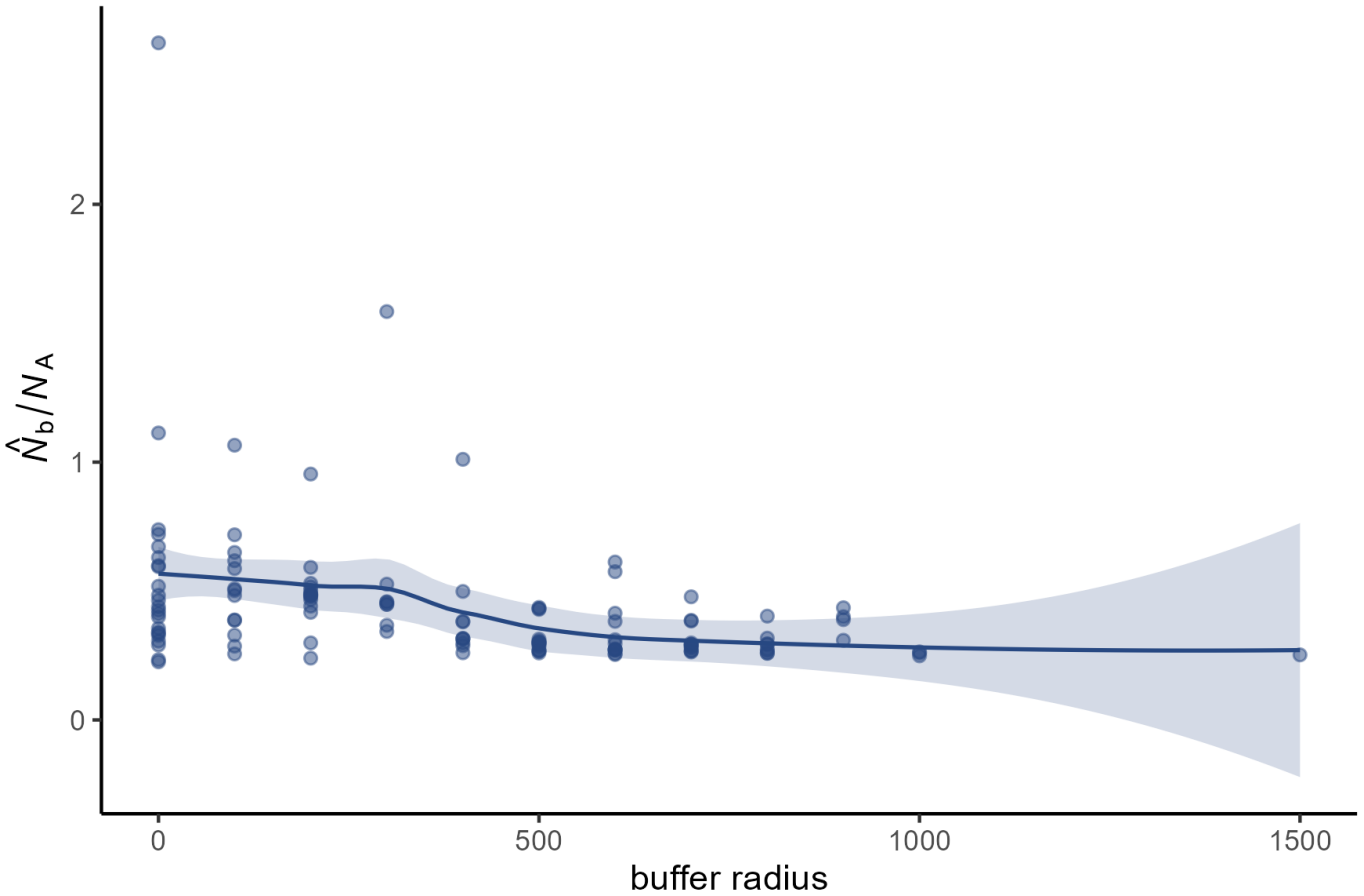
The ratio of ${\hat{N}}_{b}$, obtained using the LD method (blue), to the number of potential breeding adults (*N*_A_) in relation to buffer radius depicted using a loess curve with 95% confidence intervals (shaded area).

**FIGURE 7:**
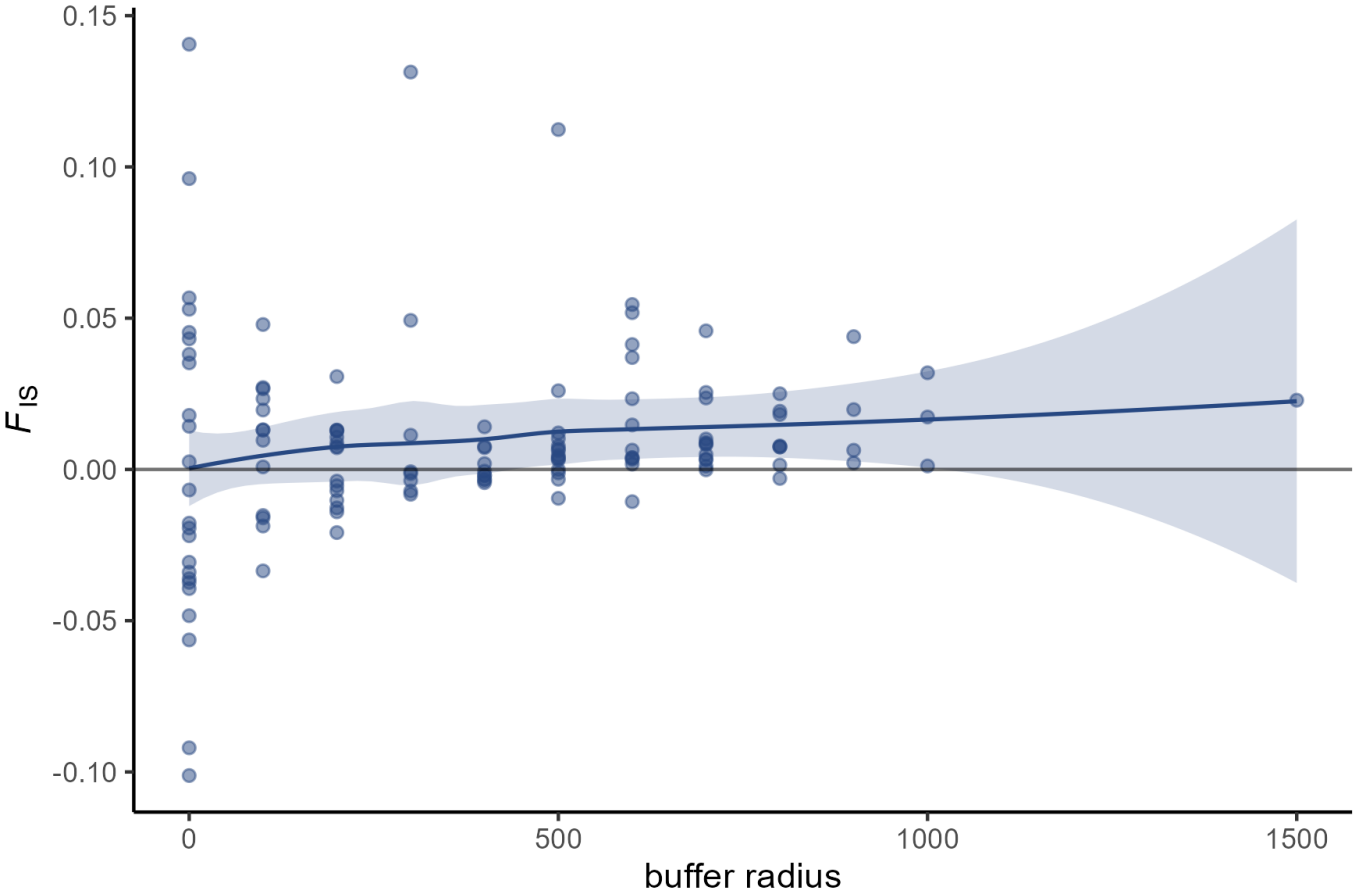
The inbreeding coefficient *F*_IS_ in relation to buffer radius using a loess curve with 95% confidence intervals (shaded area).

The ratio of the LD ${\hat{N}}_{b}$ to *N*_A_ did not reach 1 in most cases. In general, it slightly decreased with increasing buffer radius until a radius of 300 m was reached (Figure 6). From a radius of 300 m until 500 m, the ratio showed a more pronounced drop, after which it again showed a very slight decrease. This coincided with *F*_IS_ increasing with larger buffer radii (Figure 7). The 95% confidence interval did not contain zero at a buffer radius of 500 m, from which point onward *F*_IS_ values became mostly positive. The same trend was obtained when using *F*_IS_ calculated without full sibs (results not shown).

When taking the sum of all local ${\hat{N}}_{b}$ values, meta‐${\hat{N}}_{b}$ was 677 (Table 1). Since global *F*_ST_ among breeding patches was very weak, the island meta‐${\hat{N}}_{b}$ (688) differed only slightly. Another estimate of island meta‐${\hat{N}}_{b}$ was calculated with breeding patches grouped according to the local structure identified by sPCA (Figure 1). This resulted in a sum of local ${\hat{N}}_{b}$ = 509, or according to the island model in 515 (with *F*_ST_ = 0.0121, 95% CI [0.0082, 0.0173]). These estimates were all closer to the number of parents, which was 619, with of all the samples included in the analysis.

### 3.3 Random Sampling

Since spatial autocorrelation drops to zero around 460 m (Figure 2), we used this as the diameter of the breeding window. Figure S3 shows the location of the ten randomly placed buffers with a radius of 230 m. The local LD ${\hat{N}}_{b}$ that included all samples available within buffers or breeding windows (ranging from 58 to 289 samples collected in 2–8 breeding patches) had limited confidence intervals and ranged from 50 to 247 (Table S1). From a total of 100 estimates using 50 random samples within breeding windows, 54% had no finite ${\hat{N}}_{b}$ or upper confidence limit. The remaining estimates ranged from 39 to 204 (mean = 87, SD = 39). The higher the number of included breeding patches, the less likely an estimate with finite confidence limits could be obtained using 50 samples.

Assuming the population holds four neighbourhoods, we multiplied every ${\hat{N}}_{b}$ by four (excluding infinite estimates and one replicate with an undetermined value). This resulted in a mean meta‐${\hat{N}}_{b}$ = 727, which is close to the earlier obtained estimates that ranged between 600 and 700.

## 4 Discussion

In this empirical study, we investigated if general conclusions from simulations (Neel et al. 2013) of the influence of population structure on LD ${\hat{N}}_{b}$ still hold, even in other than typical continuous populations. We found that the spatially structured population showed a pattern of isolation‐by‐distance, though also a local structure consisting of groups of nearby breeding patches. Nonetheless, a downward bias on LD ${\hat{N}}_{b}$ became apparent with an increasing buffer size. The estimates started to level of at a buffer radius of 400 m, yet they were smaller than the sibship ${\hat{N}}_{b}$ at even lower radii. This corresponded with the spatial autocorrelation results, where autocorrelation was significantly positive until a maximum distance of 114 m was reached and where it hovered just above zero from 244 to 458 m. Without intermediate distance intervals, it is difficult to identify the exact maximum distance of significant positive spatial autocorrelation. Still, given our results positive autocorrelation drops to zero around 460 m in this moor frog population. Half this distance largely corresponded with the mean radius of the four general groups defining the local structure (Figure 1), and points towards the radius 2*σ* of the breeding window for a local genetic neighbourhood. Overall, it indicates that in order to estimate *N*_e_ or *N*_b_ across the metapopulation, we should take into account the presence of approximately four breeding windows.

The relationship between the ratio of LD ${\hat{N}}_{b}$ to *N*_A_ and buffer radius showed a drop at a radius of 300 m, although it was overall a negative relationship. Our estimate of the number of potential breeding adults might not be entirely appropriate to approximate the actual effective number of parents. However, the LD ${\hat{N}}_{b}$ at buffer radii ≤300 m seemed to reflect more the number of parents that produced the sample, rather than the total number of parents of the cohort of that year. Furthermore, LD ${\hat{N}}_{b}$ is influenced by both *N*_b_ and the per‐generation effective population size (*N*_e_), though can be adjusted using information on certain life history traits, such as age at maturity, variation in age‐specific fecundity and adult lifespan. With such an adjustment as proposed by Waples, Antao, and Luikart (2014), the estimate at the pond or breeding patch‐level might approach the actual effective number of parents. As we have no information on index of variation in age‐specific fecundity, adjusting the estimate with general assumptions for moor frogs of maximum life span (c. 11 years) and age at maturity (c. 3 years) would only provide slightly larger ${\hat{N}}_{b}$.

Neel et al. (2013) suggested that a signal of the Wahlund effect could be used to determine the scale of the breeding window. This is when positive *F*_IS_ values occur, as was the case in the study of mountain goats (Shirk and Cushman 2014). However, *F*_IS_ was often positive at the breeding patch level in our study. This did not change when full siblings were avoided. Nonetheless, *F*_IS_ steadily increased, on average, with increasing buffer size. This indicated the presence of the Wahlund effect, but not a specific radius at which local *N*_b_ could be confidently estimated. This is not unexpected considering the complexity of the population structure, where different levels of migration among local breeding patches exist, as suggested by pairwise *F*_ST_, and where local *N*_e_ differ among patches. Although Gilbert and Whitlock (2015) found the LD estimate of *N*_e_ to be quite accurate when sampling occurred at the local patch and when the migration rate was low, deviation of the actual *N*_e_ increased when local *N*_e_ was high (500) and migration rate was 0.1 under the island‐model migration scenario. Such high levels of local *N*_e_ do not apply to our study population. Under low levels of migration, it is, however, expected to include a component of mixture‐LD. Simulations performed by Ryman, Laikre, and Hössjer (2023) showed that when sampling two subpopulations that belong to an island model metapopulation (with 10 subpopulations and local *N*_e_ = 50), the most downward bias of *N*_e_ was obtained when migration was ≤0.2, which corresponded with an *F*_ST_ as low as 0.02. It is thus very important to obtain insight of what can be defined as local patches or subpopulations and how they relate to each other.

Due to our sampling strategy, we could maximise the number of samples at each breeding patch, without introducing a substantial amount of close relatives within patches. Still, the samples were not taken randomly and could therefore bias ${\hat{N}}_{b}$. The level of downward bias depends on the influence of migration, which could further deliver a lower estimate due to admixture‐LD or could counter the non‐random inclusion of close relatives. Without knowledge of the real *N*_b_ this is difficult to unravel.

While taking these uncertainties under consideration, our study provides further insight in the effects of spatial structure on LD ${\hat{N}}_{b}$. In spite of weak overall genetic structure (global *F*_ST_ = 0.016), the pooled set of samples underestimated ${\hat{N}}_{b}$ nearly twofold (${\hat{N}}_{b}$ = 381) than what the sum of the patch level estimates yielded (${\hat{N}}_{b}$ = 677). This bias is likely the result of mixture‐LD caused by spatial genetic structure confounding LD caused by drift. This highlights the importance of acknowledging even weak spatial genetic structure.

Also the importance of the spatial scale at which samples are taken clearly makes a large difference: in spite of the apparent homogeneity of habitats (the entire area of Klein Schietveld contains suitable terrestrial and aquatic habitat for *R. arvalis*) and the fact that the extent of the entire metapopulation is mostly within the dispersal capacity of moor frog individuals, sampling locally within a confined area (assuming that under random mating across the population the exact location of samples didn't matter) would greatly underestimate the effective size of the population (Table 1). LD $\;{\hat{N}}_{b}$ from single breeding patches were on average 28.2. Even the largest ${\hat{N}}_{b}$ for a single breeding patch (75.4, CI (50.2–128.2)) would potentially be a tenfold underestimation of the meta‐*N*_b_. Overall, this highlights how easily *N*_e_ estimates are biased, and this has further consequences for ratios of *N*_e_ to census population size (*N*_c_), which are used increasingly in biodiversity policy and management evaluation (e.g., Mastretta‐Yanes et al. 2024) and may lead to significant implications for conservation strategies. Conservation decisions based on biased estimates of *N*_e_ can lead to interventions that might not fully align with the long‐term genetic goals for a population. In this study, the LD estimate may inaccurately suggest that the moor frog population falls short of the *N*_e_ >500 threshold, which is one of the three indicators for maintaining genetic diversity proposed in the post‐2020 Global Biodiversity Framework by the Convention on Biological Diversity (CBD) (Hoban et al. 2020).

When investigating a spatially structured population, it may seem prudent to calculate LD $\hat{N}$_b_ within local patches. However, this presents challenges in obtaining a sufficient number of samples within each patch to accurately estimate local *N*_b_, as well as in sampling enough patches to gain a comprehensive understanding of the total *N*_b_. The latter is nonetheless a rough estimate and should therefore be used with caution. A simplified approach to the island model might be to consider the relationship between neighbourhood size *N*_n_ and meta‐*N*_e_ (or here *N*_b_ and meta‐*N*_b_, respectively) when the *F*_ST_ is small. When *F*_ST_ is very small, like here, the effect of genetic structure on meta‐*N*_e_ can be negligible. Based on the distribution of *R. arvalis* at the time of sampling and the size of the breeding window (*r* = 230 m), it is reasonable to assume approximately four breeding windows, more or less corresponding to the observed spatial structure (Figure 1). Our random sampling scheme using randomly placed potential breeding windows and employing 50 samples each, a number regularly used in empirical studies, yielded variable local LD ${\hat{N}}_{b}$ with often unbounded confidence intervals, particularly when >4 breeding patches were included. Additionally, local estimates with limited confidence intervals tended to bias meta‐*N*_b_ downwardly. To improve accuracy, sampling efforts should probably encompass numerous breeding patches and a higher number of samples to better approximate local *N*_b_ within a breeding window, particularly in a large population such as the one included in this study. In spite of these limitations, we can still extract useful information from this exercise. Overall, >90% of the population occurs within the area of four breeding windows (Figure S1). Under the assumptions that the density is equal across all breeding windows and that genetic structure (*F*_ST_) is too weak to affect meta‐*N*_e_, we tried to verify to what extent we can estimate meta‐*N*_e_ by extrapolating from a single breeding window. Averaging over replicates multiplied by four neighbourhoods provided an estimate of meta‐*N*_b_ that was close to earlier estimates using all samples available (between 600 and 700). Likewise, if we utilise all available samples within a single breeding window (buffers a‐j in Table S1, total values) and estimate 4${\hat{N}}_{b}$, the average meta‐${\hat{N}}_{b}$ = 604 (range 200–990) is reasonably close to the number of parents calculated for the entire sample. This corroborates the idea that it is possible to estimate the meta‐${\hat{N}}_{b}$ by extrapolating from individual neighbourhood ${\hat{N}}_{b}$, reducing the need for extensive sampling of similar metapopulations, such as other pond‐breeding amphibians with weak genetic structure, as we have done in this study.

Despite the presumed unknown spatial structure in the random sampling exercise, accounting for the radius of the breeding window was considered. Nonetheless, broader sampling would still be necessary to estimate the mean size of a neighbourhood accurately. Alternatively, sampling multiple though a limited number of ponds across the population and using the mean local ${\hat{N}}_{b}$ at the pond‐level, multiplied by the number of ponds where moor frog is present, could yield meta‐${\hat{N}}_{b}$. Although this approach carries the risk of over‐ or underestimating meta‐*N*_b_, it provides insight into potential substructure and global *F*_ST_. Further validation through additional testing is warranted for both approaches and in other similar systems.

## Conflicts of Interest

Mergeay, Joachim is an Editorial Board member of *Evolutionary Applications* and a co‐author of this article. To minimise bias, they were excluded from all editorial decision‐making related to the acceptance of this article for publication.

## Supporting information


Data S1.

## Data Availability

Data Availability Statement sampling locations and microsatellite genotypes are accessible at Dryad Digital Repository: https://doi.org/10.5061/dryad.j0zpc86ps.
