# Supplementary material for: Dealing With Assumptions and Sampling Bias in the Estimation of Effective Population Size: A Case Study in an Amphibian Population
Source: Evol Appl. 2024 Sep 13;17(9):e70015. doi: 10.1111/eva.70015 (PMC11393452; doi:10.1111/eva.70015)
Supplement: Supplementary file 1 — Data S1. [file EVA-17-e70015-s001.pdf]

Supporting Information

Title: Dealing with assumptions and sampling bias in the estimation of effective population size: a case study in an amphibian population

Karen Cox, Sabrina Neyrinck, Joachim Mergeay

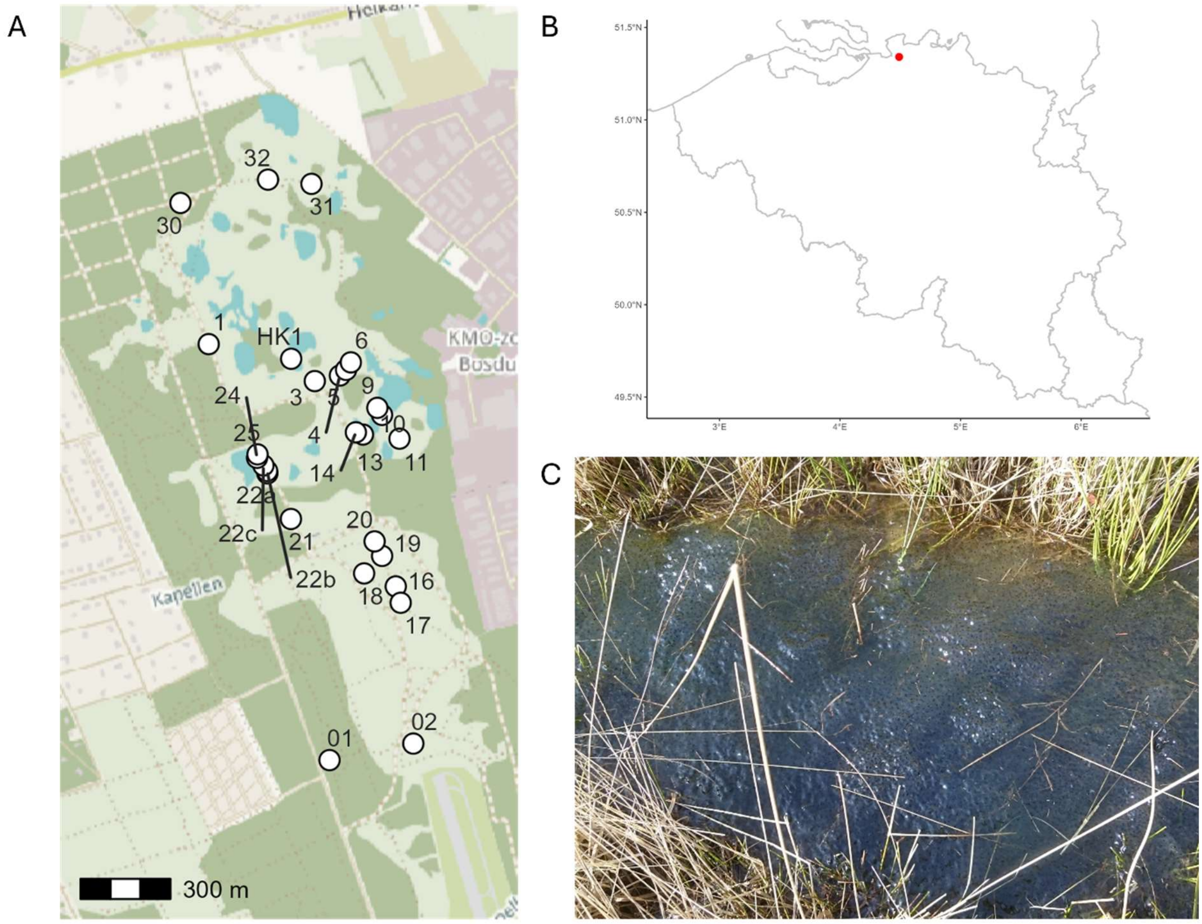

FIGURE S1
The breeding patches (white circles with corresponding IDs) where egg clutches of *Rana arvalis* and *R. temporaria* were sampled (A), situated in Klein Schietveld (red dot) located in the north of Belgium (B; with country borders indicated in grey). The photograph (C) shows drifting egg clutches. Base map in A: © OpenStreetMap contributors.

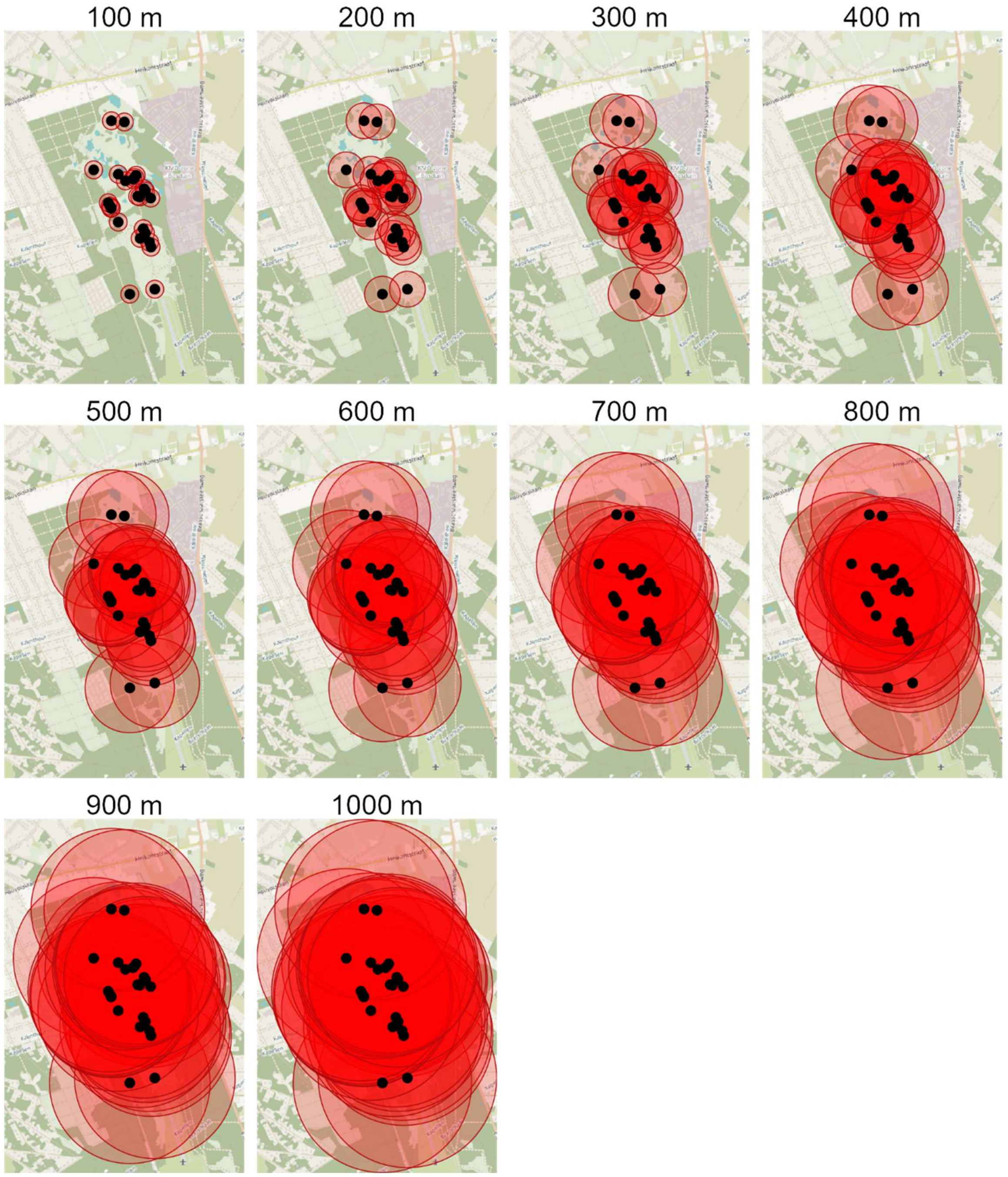

FIGURE S2
Plots of buffers (red circles) placed around the locations of the sampled breeding patches of moor frog (*Rana arvalis*) (black dots) with the radius length mentioned above each plot. Base map: © OpenStreetMap contributors.

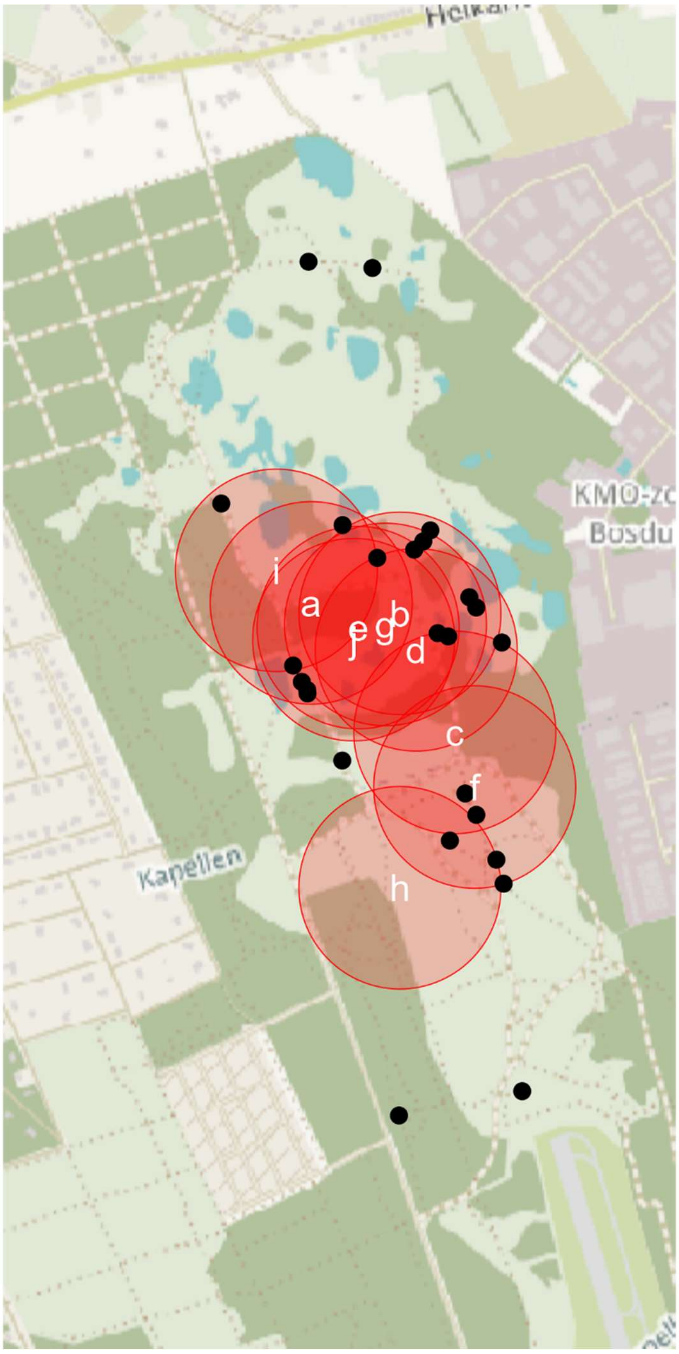

FIGURE S3
Ten randomly placed buffers with a radius of 230 m (red circles) indicated with a white letter in each centre. Black dots are the sampled breeding patches of moor frog. Base map: © OpenStreetMap contributors.

TABLE S1. Resulting LD $\widehat{N}_b$ and 95% confidence intervals when using randomly placed buffers with a radius of 230 m. Buffer ID corresponds with the respective buffer shown in Fig. S3. In each buffer or breeding window 50 genotypes were sampled randomly and this was repeated 10 times (set). An estimate of LD $\widehat{N}_b$ was also given for all samples within the buffer. N samples: number of samples included in the estimation; N patches: number of breeding patches included.

Buffer ID	set	N samples	N patches	LD $\widehat{N}_b$ (95% CI)
a	1	50	6	98.7 (53.4-334.2)
a	2	50	5	132.9 (52.8-∞)
a	3	50	5	226.3 (83.5-∞)
a	4	50	6	87.4 (42.2-520.2)
a	5	50	5	95.5 (48.8-422.9)
a	6	50	6	165.3 (65.4-∞)
a	7	50	5	118.9 (43.3-∞)
a	8	50	6	250.4 (66.9-∞)
a	9	50	5	159.4 (66.1-∞)
a	10	50	6	86.4 (42.6-439.3)
a	total	166	6	136.1 (99.6-198.6)
b	1	50	8	377.5 (67.2-∞)
b	2	50	8	368.5 (114.0-∞)
b	3	50	8	198.8 (62.4-∞)
b	4	50	8	69.5 (30.5-702.1)
b	5	50	8	116.0 (49.9-∞)
b	6	50	8	327.6 (90.2-∞)
b	7	50	7	102.6 (48.7-837.9)
b	8	50	8	161.8 (74.3-33230.4)
b	9	50	8	253.8 (61.8-∞)
b	10	50	8	∞ (232.9-∞)
b	total	289	8	247.4 (183.5-355)
c	1	50	4	89.5 (45.9-373.9)
c	2	50	4	106.6 (48.3-2119.4)
c	3	50	4	62.3 (32.7-198.8)
c	4	50	4	129.3 (58.1-19908.6)
c	5	50	4	85.5 (43.5-365.5)
c	6	50	4	105.5 (48.9-1252.9)
c	7	50	4	85.9 (42.6-423.3)
c	8	50	4	207.5 (88.0-∞)
c	9	50	4	146.7 (67.6-6439.9)
c	10	50	4	104.9 (48.5-1294.8)
c	total	111	4	98.1 (62.1-183.1)
d	1	50	7	145.8 (48.9-∞)
d	2	50	7	683.1 (103.8-∞)
d	3	50	7	∞ (169.8-∞)
d	4	50	7	925.9 (115.6-∞)
d	5	50	7	124.5 (48.4-∞)
d	6	50	7	∞ (144.8-∞)

Buffer ID	set	N samples	N patches	LD $\widehat{N}_b$ (95% CI)
d	7	50	7	377.2 (84.1-∞)
d	8	50	7	213.2 (77.4-∞)
d	9	50	7	184.3 (63.9-∞)
d	10	50	7	∞ (120.6-∞)
d	total	213	7	218.2 (151.1-352.1)
e	1	50	8	394.9 (96.4-∞)
e	2	50	8	∞ (141.1-∞)
e	3	50	8	202.5 (72.8-∞)
e	4	50	8	83.6 (46.3-244.5)
e	5	50	8	335.8 (104.9-∞)
e	6	50	8	142.2 (111.2-∞)
e	7	50	8	548.1 (120.9-∞)
e	8	50	8	172.1 (64.3-∞)
e	9	50	8	204.3 (99.4-2991.3)
e	10	50	8	271.2 (82.8-∞)
e	total	266	8	213.5 (156.5-310.7)
f	1	50	5	217.6 (71.5-∞)
f	2	50	5	136.6 (60.1-∞)
f	3	50	5	127.3 (58.9-2472.9)
f	4	50	5	80.7 (42.0-299.3)
f	5	50	4	83 (40.5-436.1)
f	6	50	5	314.5 (85.8-∞)
f	7	50	5	256.8 (112.4-∞)
f	8	50	5	116.4 (51.8-12208.4)
f	9	50	5	124.0 (63.5-632.1)
f	10	50	5	119.1 (58.6-804.5)
f	total	184	5	104.2 (73.3-157.6)
g	1	50	7	206.6 (77.9-∞)
g	2	50	7	146.9 (62.6-∞)
g	3	50	7	16474.1 (132.9-∞)
g	4	50	7	529.4 (116.6-∞)
g	5	50	7	119.7 (57.4-1068.7)
g	6	50	7	282.7 (76.6-∞)
g	7	50	7	398.2 (90.9-∞)
g	8	50	7	399.0 (104.5-∞)
g	9	50	7	289.9 (79.3-∞)
g	10	50	7	441.3 (115.1-∞)
g	total	207	7	214.7 (148.9-345.7)
h	1	50	2	61.6 (38.8-119.6)
h	2	50	2	64.9 (37.9-151.2)
h	3	50	2	66.5 (32.4-298.2)
h	4	50	2	48.2 (28.4-103.6)
h	5	50	2	51.7 (28.9-127.9)
h	6	50	2	58.0 (32.1-151.5)
h	7	50	2	74.5 (47.7-143)

Buffer ID	set	N samples	N patches	LD $\hat{N}_b$ (95% CI)
h	8	50	2	141.0 (65.2-4055.5)
h	9	50	2	80.9 (44.6-238.6)
h	10	50	2	90.2 (46.8-354)
h	total	119	2	66.9 (47.4-99.9)
i	1	50	3	41.8 (26.0-79.0)
i	2	50	3	49.4 (32.0-88.0)
i	3	50	3	52.0 (33.9-92.7)
i	4	50	3	45.7 (28.6-86.0)
i	5	50	3	42.3 (25.6-83.9)
i	6	50	3	52.1 (30.8-112.9)
i	7	50	3	48.3 (28.0-108.2)
i	8	50	3	38.8 (25.4-65.9)
i	9	50	3	49.9 (28.2-118.9)
i	10	50	3	54.5 (32.5-116.8)
i	total	58	3	50.0 (33.1-84.8)
j	1	50	7	490.6 (126.4-∞)
j	2	50	7	∞ (176.6-∞)
j	3	50	7	321.1 (101.1-∞)
j	4	50	7	143.2 (55.4-∞)
j	5	50	7	138.9 (54.9-∞)
j	6	50	7	306.2 (96.1-∞)
j	7	50	7	91.2 (47.0-375.1)
j	8	50	7	191.1 (79.8-∞)
j	9	50	7	132.2 (55.8-∞)
j	10	50	7	146.6 (65.8-∞)
j	total	224	7	162.0 (117.7-237.4)
